# Respiratory Diseases Diagnosis Using Audio Analysis and Artificial Intelligence: A Systematic Review

**DOI:** 10.3390/s24041173

**Published:** 2024-02-10

**Authors:** Panagiotis Kapetanidis, Fotios Kalioras, Constantinos Tsakonas, Pantelis Tzamalis, George Kontogiannis, Theodora Karamanidou, Thanos G. Stavropoulos, Sotiris Nikoletseas

**Affiliations:** 1 Computer Engineering and Informatics Department, University of Patras, 26504 Patras, Greecetsakonas_k@ac.upatras.gr (C.T.); g.kontogiannis@ac.upatras.gr (G.K.); nikole@ceid.upatras.gr (S.N.); 2 Pfizer Center for Digital Innovation, 55535 Thessaloniki, Greece; theodora.karamanidou@pfizer.com (T.K.); thanos.stavropoulos@pfizer.com (T.G.S.)

**Keywords:** respiratory symptoms, respiratory disease, audio analysis, signal processing, machine learning, digital biomarkers, audio-based biomarkers, systematic review

## Abstract

Respiratory diseases represent a significant global burden, necessitating efficient diagnostic methods for timely intervention. Digital biomarkers based on audio, acoustics, and sound from the upper and lower respiratory system, as well as the voice, have emerged as valuable indicators of respiratory functionality. Recent advancements in machine learning (ML) algorithms offer promising avenues for the identification and diagnosis of respiratory diseases through the analysis and processing of such audio-based biomarkers. An ever-increasing number of studies employ ML techniques to extract meaningful information from audio biomarkers. Beyond disease identification, these studies explore diverse aspects such as the recognition of cough sounds amidst environmental noise, the analysis of respiratory sounds to detect respiratory symptoms like wheezes and crackles, as well as the analysis of the voice/speech for the evaluation of human voice abnormalities. To provide a more in-depth analysis, this review examines 75 relevant audio analysis studies across three distinct areas of concern based on respiratory diseases’ symptoms: (a) cough detection, (b) lower respiratory symptoms identification, and (c) diagnostics from the voice and speech. Furthermore, publicly available datasets commonly utilized in this domain are presented. It is observed that research trends are influenced by the pandemic, with a surge in studies on COVID-19 diagnosis, mobile data acquisition, and remote diagnosis systems.

## 1. Introduction

Respiratory diseases mainly affect the lungs and, consequently, the vocal cords, leading to changes in an individual’s voice timbre. The sounds produced during coughing and breathing, known as respiratory sounds, can provide valuable information for the identification and diagnosis of respiratory diseases. With the continuous improvement of technology, these sounds can be easily recorded nowadays with minimal invasiveness, making them effective diagnostic tools [1]. Moreover, in recent years, advances in ML have also enabled the development of software tools capable of the automated analysis of respiratory sounds. These tools employ algorithms to examine the sound signals generated by the human respiratory system, identifying patterns indicative of specific diseases.

Several attributes of respiratory sounds contribute to the diagnosis of respiratory diseases. For instance, concerning the upper respiratory sounds, the type (dry or wet) and the duration of the cough could indicate a possible respiratory disease [2]. On the other hand, in the lower respiratory, the presence of crackles, wheezes, and stridor in respiratory sounds often suggests that an underlying respiratory disease may be present. In addition, voice and speech sounds offer important clues regarding respiratory diseases. For example, voice pitch and volume changes could indicate an early sign of conditions such as laryngeal carcinoma and vocal cord dysfunction [3]. ML algorithms can leverage these sound characteristics, analyze the changes, and provide diagnostic tools for such conditions.

In recent years, research on the cough and respiratory sounds classification has gained momentum, particularly in 2020 and 2021 due to the emergence of the COVID-19 pandemic. As a direct consequence of the spread of the COVID-19 respiratory disease, researchers directed their focus on the classification of body sounds, which could be useful biomarkers of the disease. Regarding the cough domain, two main sub-problems arose. The first pertains to the differentiation of coughs based on the underlying conditions such as Asthma, chronic obstructive pulmonary disease (COPD), and COVID-19 [4,5,6], whereas the second is related to the identification of individual cough instances in a voice recording [7]. As far as respiratory sound classification is concerned, the field has significantly less attraction but great popularity nonetheless. The current focus is on replacing the standard auscultation procedure performed by the doctor with remote auscultation utilizing an IoT device [1].

It is worth noting that the integration of ML and Artificial Intelligence (AI) into the analysis of these sounds also allows for revolutionizing, in a groundbreaking manner, the identification and diagnosis of respiratory diseases. AI leads to the development of automated intelligent systems, which in turn, offer quick and accurate diagnoses, reducing the need for invasive procedures, enabling early treatment of respiratory conditions, and providing continuous monitoring of the patient, even remotely. As a result, these systems have the potential to reduce the burden on healthcare systems by providing diagnosis-assistive tools for medical experts as well as valuable information and data to stakeholders that can influence healthcare on a large scale.

However, there are still several limitations regarding automated and effective respiratory disease identification. Therefore, the primary objective of this literature review is to summarize recent studies, identify the current approaches and trends, and pave the way for future advancements in the domain. Three types of respiratory audio biomarkers were identified to be the most commonly used among studies for the identification of respiratory diseases and patient health monitoring with the use of ML techniques. These are cough sounds, respiratory sounds, and voice/speech sounds. Consequently, this three-in-one review exclusively focuses on each biomarker area separately, examines the relevant studies, and provides a critical presentation of their findings.

*Research Questions*. Specifically, the main research questions we examined that led us to conduct this systematic review, are the following: (a) what are the trends in respiratory disease identification over the last 5 years, and which are the domain-specific studies, (b) is it feasible to detect respiratory diseases or their symptoms using machine learning-based methods, digital signal processing, and feature engineering, and (c) what are the most prominent methods and datasets for the respiratory diseases’ identification? In response to those questions, we carried out our study, which answers the aforementioned concerns. Specifically, answers to (a) and (b) are intended for business, healthcare providers, research, and policy-makers, while answering question (c) via our research, we pave the path for ML research regarding the specific domain of smart respiratory disease identification.

The remaining sections of this document are organized as follows: Section 2 outlines the methodology employed for selecting the studies included in this review. Section 3, Section 4 and Section 5 present the findings related to each audio biomarker, respectively. Next, Section 6 describes the publicly available datasets that are most frequently utilized in the studies reviewed. Moreover, Section 7 offers an overview of the findings and discusses our remarks, and finally, Section 8 concludes the review.

## 2. Methods

To select relevant studies, we utilized Google Scholar and PubMed as the primary resources. Due to the various variations of respiratory diseases, a single query would yield a substantial number of studies. Moreover, studies with low relevance to the topic could be collected, as similar terminology is also used in purely medicine-related studies. Therefore, queries were constructed for each of the signature respiratory sounds, and the research results were derived from papers published until 30 November 2022.

The first signature sound commonly used for respiratory disease identification and diagnosis is a cough. Numerous studies on cough recognition as well as disease identification from cough sounds exist. To include studies from both sub-topics, a single search was performed on the mentioned search engines, using various keywords such as “cough detection”, “cough classification”, “cough sounds”, “machine learning”, and other performance-related keywords like “specificity”, “accuracy”, and “sensitivity”. The 1st query in the code snippet below corresponds to the search conducted for cough-related studies and is structured to cover all possible combinations of the specified words.

1. ("cough detection" OR  "cough classification" OR "cough sounds"
 OR "cough audio" OR "cough analysis")
 AND
("specificity" OR "accuracy" OR "sensitivity" OR "f1"
 OR "true positive" OR "AUC" or "MFCC")
 

Breath and lung sounds on the other hand, whether captured by stethoscopes, microphones, or mobile devices, constitute another set of signature sounds relevant to respiratory diseases. These sounds mainly correspond to symptoms from the lower respiratory tract. Again, in this case, the same search engines were utilized, employing keywords such as “respiratory disease classification”, “lung sound classification”, “respiratory sound”, “machine learning”, and performance-related keywords similar to those used for the cough search. Despite the specificity of individual searches, a significant number of studies were returned. The 2nd query related to that subject can be seen in the code snippet below.

2. ("respiratory disease classification"
 OR "respiratory sound classification"
 OR "lung sound classification" OR "respiratory sound")
 AND
("ML" OR "machine learning")
 AND
("accuracy" OR "AUC" OR "f1" OR "sensitivity")
 

Last but not least, the human voice is also one of the biomarkers that can be used in the identification and diagnosis of respiratory diseases. However, creating a query was deemed non-optimal due to the extensive use of the human voice in the identification of various diseases using machine-learning techniques. Consequently, a manual search was conducted to identify appropriate studies that are relevant to our review topic.

After gathering the results from the search queries, a filtering phase was employed. Duplicate results were removed, and the analysis primarily focused on full papers. Additionally, studies published prior to 2017 were excluded. Furthermore, studies with a minimal number of citations were excluded, as this indicates a potential lack of proper evaluation by the research community. Following the filtering phase, the final set of studies included in our review was constructed by manually assessing titles, abstracts, and conclusions. Through a rigorous process, highly informative and significant studies were deliberately selected.

**Figure 1:**
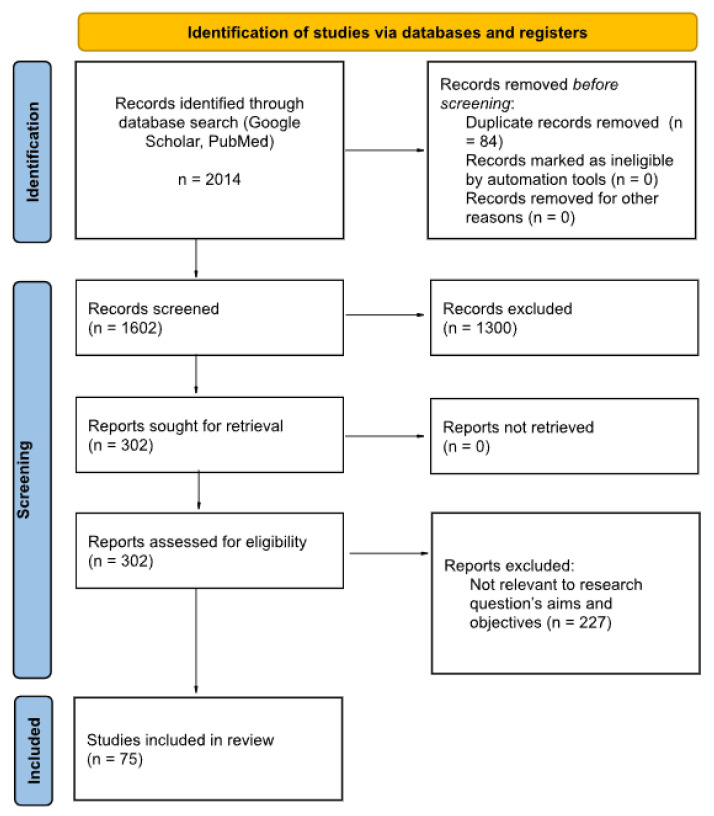
Review process flowchart.

**Figure 2:**
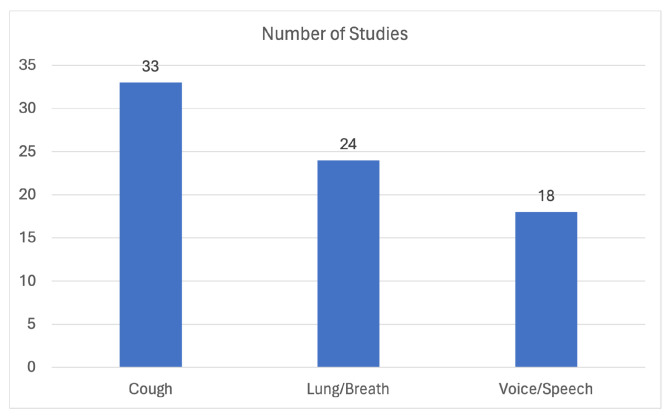
Domain distribution of the included studies.

The flowchart in Figure 1 describes the sources, numbers, and fates of all identified and screened records in this review. After all the filtering processes a total of 75 studies were found to be eligible to be added in this review. The distribution of these publications based on the domain of interest is illustrated in Figure 2.

**Figure 3:**
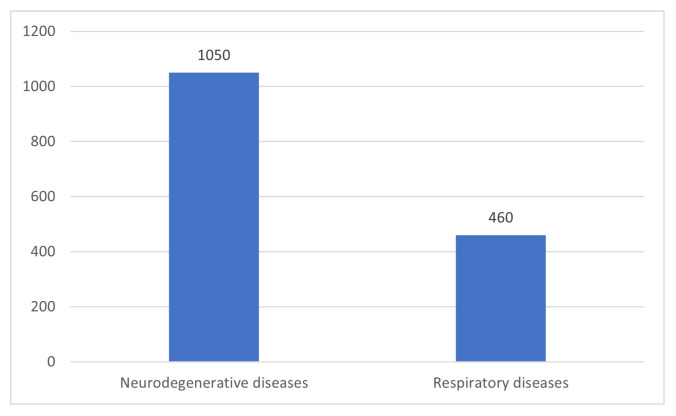
Number of neurodegenerative studies vs. number of respiratory studies.

To provide insight into the retrieval of low-relevance studies, Figure 3 compares the available studies on the classification of neurodegenerative diseases and respiratory diseases using voice recordings and ML. Specifically, the 3rd query, which can be seen below relates to the retrieval of the neurodegenerative studies. It is observed that there is a significant difference between the number of studies that are related to neurodegenerative diseases’ identification compared to the ones that correspond to respiratory diseases. In particular, the number of studies that are published during the same time period akin to neurodegenerative diseases is almost double those that are relevant to respiratory diseases’ analysis.

3. ("Alzheimer" OR "Parkinson’s disease" OR "bradykinesia")
 AND
("speech signal processing" OR "speech sounds"
 OR "Voice Recognition" OR "speech classification")
 AND
("classification" OR "accuracy")
 AND
("machine learning" OR "deep learning")
 

**Figure 4:**
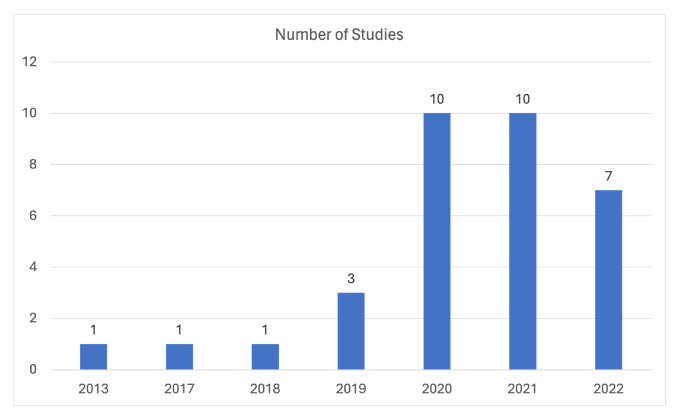
Distribution of cough-related studies across publication years.

Regarding the cough domain, Figure 4 presents a visual depiction of the distribution of the included studies across different publication years, providing a graphical representation of the distribution of studies across each different publishing year. Notably, there is an observable increasing trend in the number of domain-related studies, which can be attributed to the ongoing COVID-19 pandemic. Additionally, it is worth mentioning that, although some studies may currently have relatively low citation counts, there appears to be a promising trend indicating potential future citations, considering the review’s scope extends up until November 2022.

**Figure 5:**
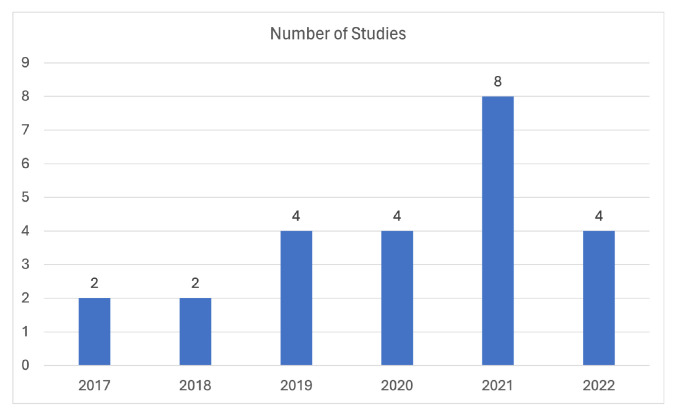
Distribution of respiratory sounds related studies across publication years.

In the respiratory sounds classification domain, Figure 5 provides a graphical representation of the distribution of studies included in this review based on their respective publication years. The Figure illustrates a consistently growing and sustained interest in this topic over the past few years, indicating a continuous and stable research focus in this area since 2019.

**Figure 6:**
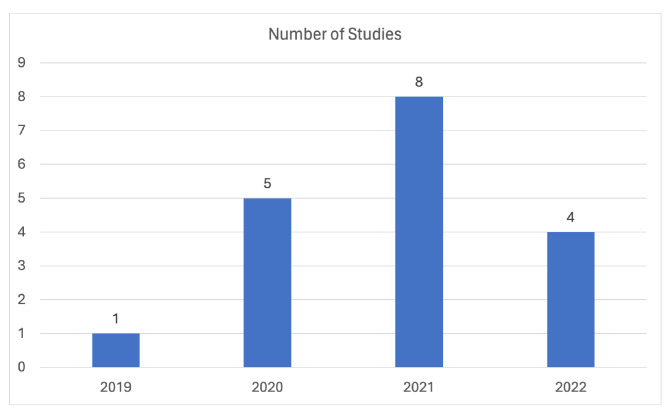
Distribution of voice/speech analysis related studies across publication years.

Finally, Figure 6 visually presents the distribution of studies related to voice/speech analysis for the identification of respiratory diseases, categorized based on their respective publication years, within the context of this review. Notably, there is a noticeable rise in the number of related studies in 2021, possibly attributed to the COVID-19 pandemic, which likely spurred increased research focus on respiratory disease identification and related voice/speech analysis.

## 3. Cough Sounds Analysis for Upper Respiratory Symptoms

In this Section, a review of the analysis of cough sounds and the outcomes that this process yields using ML methodology takes place. In particular, we start by describing how the proper datasets were created that were used by the corresponding studies for automated intelligent analysis. Afterward, the focus of these studies regarding the analysis of the sounds or the identification of diseases is outlined, and subsequently, the algorithmic approach for both data processing and AI modeling of how the studies conducted their analysis is described and some significant results are presented. Table 1 summarizes the studies that are presented in this Section.

### 3.1. Data Acquisition

The automated detection and classification of cough sounds is a well-researched domain within the scientific community, particularly gaining significant research attention during the recent period of COVID-19 pandemic exacerbation. The discernibility of cough sound events enables their capture using commonly available equipment, such as smartphone and web camera microphones, without the requirement for specialized or high-end recording devices.

In response to the growing demand for cough sound datasets, several open-source repositories have emerged, especially during the pandemic, offering valuable resources for research and development. Notable examples include the Virufy [37], the COVID-19 Sounds [38], the Coswara [39], the Corp [40], and the NoCoCoDa [41] dataset. These datasets encompass a diverse range of cough sounds captured from individuals with various underlying respiratory diseases, primarily focusing on COVID-19 cases, as well as from healthy volunteers. For the datasets’ proper creation, it was also deemed necessary for the participants to possess different demographic information. The recordings are obtained through smartphones, web applications, and high-end recording devices. Furthermore, the immediate availability of these well-documented and representative real-world data has served as a catalyst, spawning numerous in-depth studies within the field of cough detection and classification, such as [7,10,12,14,16,17,18,19,28,31,32,34,35], which have achieved notable results.

In greater detail, the Virufy dataset, which is openly accessible, contains cough sounds that were acquired through two distinct approaches: (a) the collection of cough sounds in a hospital setting, overseen by medical professionals, and (b) the aggregation of crowd-sourced cough sounds obtained via smartphones and web camera microphones from diverse geographic regions. This dataset has been used in several studies such as [12,19,32,34].

The COVID-19 Sounds, a large-scale open-source (upon request) audio dataset, comprises recordings of coughs, breathing, and voices. Each sample within this dataset is accompanied by metadata files that provide crucial information about individual recordings, including relevant descriptors and the respiratory health status of the respective volunteer. For their research purposes, the authors of [19,35] have utilized this dataset.

Furthermore, the Corp dataset stands as a thoroughly designed, collected, and annotated open-source audio dataset. This dataset consists of 168 h of cough sound recordings obtained from 42 patients diagnosed with various respiratory diseases. Its comprehensive nature provides researchers with a valuable resource for studying cough classification and detection, enabling insights into diverse respiratory conditions.

Lastly, the NoCoCoDa dataset, a publicly accessible cough sound database, comprises a collection of 73 distinct cough events. This dataset has been utilized in studies such as [19,34].

**Figure 7:**
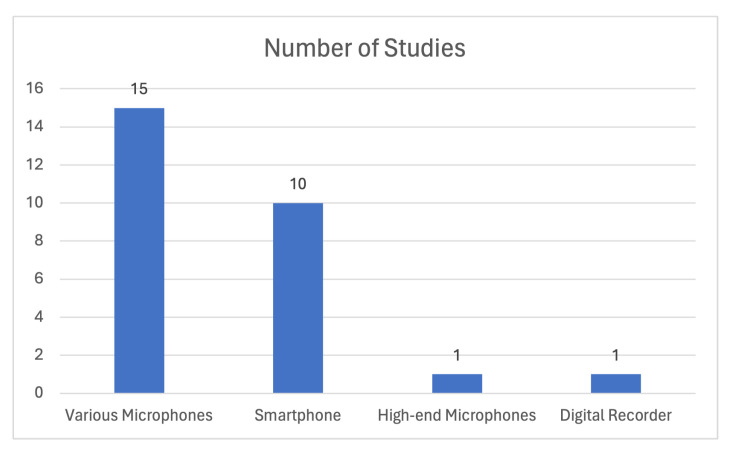
Distribution of cough-related studies with respect to the device used for data acquisition.

It should be noted that several studies, such as [4,5,6,8,9,11,15,20,21,22,23,24,25,26,27,29,30,33,36], have developed their own proprietary datasets explicitly tailored to their respective research objectives. In more detail, the authors of [4,8,21,23,24,26,27,30,36] employed smartphones to record cough sounds from their participant cohorts. On the other hand, high-end recording devices were utilized for cough sound data acquisition in studies such as [8,20,29,33]. Figure 7 visually represents the distribution of studies based on the recording devices used for data acquisition. The Figure illustrates an ongoing trend in the utilization of readily available hardware, such as smartphone microphones, for capturing cough sounds in the research on this subject.

### 3.2. Objectives in Cough Sound Analysis Studies

There are primarily two categories of research papers focused on the utilization of cough sounds. The first category pertains to the detection of cough sounds, involving the identification of a sound signal as a cough. Within this category, various authors [7,14,17,18,21,22,23,24,25,28,29] have achieved detection accuracies as high as 99.64%.

The second category concerns the classification of cough sounds with respect to the underlying respiratory disease of the patient, as well as the identification of cough types such as wet or dry coughs [20,26]. Coughing is a common symptom of many respiratory diseases, and variations in cough sounds can provide insights into the health status and specific respiratory conditions of individuals. For instance, in [8] the authors collected cough sound samples from patients with asthma, COPD, Interstitial lung disease (ILD), bronchitis, and pneumonia and achieved a cough classification accuracy of 93%. COPD was the disease of interest for the authors of [5] as well, in which a 0.89 ROC curve in correctly identifying coughs produced by patients with the disease was demonstrated. Similarly, in [26] an 88% accuracy in classifying cough sounds related to COPD was achieved. COVID-19, pertussis, and bronchitis cough sounds were classified with an accuracy of 92.64% in [14] while [15] achieved an impressive accuracy of 95.04% encompassing asthma, bronchitis, and COVID-19 cough sounds. Asthma was also a prominent disease of interest for the authors of [4,26,36] as well. Finally, the authors of [30] focused on Croup as their main disease of interest, reaching an accuracy of 86.09%, while in [33] the authors achieved an accuracy of 84.54% in identifying cough sounds produced by patients with Tuberculosis (TB).

COVID-19 stands out as the most extensively studied disease in a significant portion of the literature. The abundance of COVID-19-related datasets, the need for the acceleration of testing purposes for disease prevention, and the monitoring of the severity of the disease have collectively contributed to the proliferation of relative studies. Several authors [6,9,10,11,12,14,27,31,34,35] have proposed novel algorithmic methods for COVID-19 identification through cough sounds, achieving highly satisfactory results. The reported accuracy across these studies ranges from 70% to an impressive 99%.

**Figure 8:**
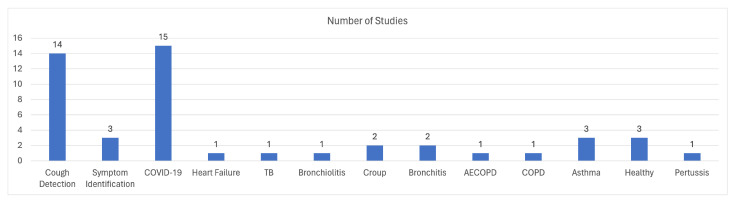
Distribution of studies with respect to their research topic in the cough area.

In conclusion, Figure 8 illustrates the distribution of the studies related to cough sounds, categorized based on their research topics, as they were described above.

### 3.3. Implementation Approach

The algorithmic approaches employed in the aforementioned studies exhibit significant variations and are heavily reliant on the training data utilized. Cough detection inherently involves the classification of audio segments into either cough or non-cough events, making it a form of cough classification. Consequently, the algorithms utilized for these objectives often share common attributes. Similar to other time-series-related ML problems, a well-defined framework needs to be followed to achieve desirable results. This framework typically consists of three sequential stages: (a) the data preparation stage, which is aimed at manipulating the raw signal by filtering, denoising, splitting the input into equal segments, windowing, and other similar techniques; (b) the Feature Extraction phase, in which a set of features that are providing descriptive information about the cough event is extracted from the input signal; and (c) the classification stage, where the classification of the input signal occurs. These stages are followed by most of the studies presented. Deep Learning-based solutions slightly deviate from this framework, as the Feature Extraction stage is performed automatically with the classification stage by the neural networks mitigating that way the need for explicitly defining features.

During the data preparation stage, a challenge that most studies attempt to tackle is the isolation of cough events from the raw signal. Studies suggest a variety of methods for labeling the raw signals such as utilizing a moving window signal deviation as a function of time [8] and splitting the signal based on the silence segments [15], manual labeling, by using software such as PRAAT [17] and others [31], or even empirically [4,7,10,16,18,20,23,26,28,29,30,36]. Among other methods are classifiers that are able to distinguish the energy levels among different segments of a signal [12] and finally, Empirical Mode Decomposition (EMD) [11]. Additionally, a variety of prepossessing steps such as filtering [15,23,26], downsampling [4,11,15,16,25,26,36], and normalizing [26,31] are being incorporated by the authors before Feature Extraction.

According to the data engineering phase, in the domain of sound analysis and recognition, it is customary to transform the raw input signals into a feature representation before utilizing them in a model. One of the most widely adopted techniques in audio processing and pattern recognition is the extraction of Mel Frequencies Cepstral Coefficients (MFCCs). Interestingly, among the 32 studies examined, 25 of them employed the MFCCs method for extracting cepstral features and utilized them as inputs to a classifier, often in combination with other features. MFCCs can be employed in two distinct categories to represent a signal. Firstly, they can be manually engineered as features, where a feature matrix is created based on the extracted MFCCs. Alternatively, the signal can be transformed directly into an MFCCs representation, circumventing the need for explicit feature engineering. Specifically, in [8,12,15,16,19,20,21,24,25,30,31,33,34] the authors extract MFCCs features along with other common features for audio processing such as Zero-Crossing Rate (ZCR), spectral centroid, spectral bandwidth, spectral roll-off, spectral flux, entropy, kurtosis, and log-filterbank energies. In these studies, the MFCCs are hand-engineered. Studies such as [9,10,11,17,35] use the full MFCCs representations of the signals as input to their learning models in combination with other features and representations.

In Refs. [18,26], popular software (e.g., openSMILE [42]) is utilized to extract a diverse set of features, which are subsequently subjected to feature selection techniques to retain only the most significant ones for the final feature matrix. Conversely [29], primarily employs Fast Fourier Transform (FFT) to extract features from the cough signals. Then, the authors apply Principal Component Analysis (PCA) to reduce the dimensionality of the resulting feature vectors. However, several other studies [7,14,23,28] opt to use Mel-Spectrograms as inputs to their model architectures. Additionally [27], proposes an innovative approach to Feature Extraction by utilizing a DNA pattern feature generator, while [15] introduces a novel method for representing signals using embeddings.

In the classification stage, the choice of model architecture varies among studies, as it is highly dependent on the authors’ objectives. Studies can be divided into two main categories based on the selected models. The first category utilizes ML models which are based on the statistical learning theory such as Support Vector Machines (SVM) [12,21,24,25,30,31,34], *K*-NN [19,21,25,27,31,34], LRM [19,24,30,33], Random Forest (RF) [8,12,21,26], XGboost [6,8,19], and others. The second category utilizes Artificial Neural Networks (ANNs) like 2D-Convolutional Neural Networks (CNNs) [7,10,11,14,17,23,28], and LSTM (RNN) architectures [13]. Refs. [9,10,31] follow a very interesting approach, where the authors utilize state-of-the-art architectures such as a 3 ResNet-50 placed in parallel architecture, a VGG-16, and a ResNet-50, respectively. ANNs are used in [29] while in [16,31,33] both ML models that are related to statistical learning theory and ANNs are tested during experimentation.

It is also worth mentioning that several research studies focused on cough classification explore multi-modal approaches, typically consisting of two stages. The first stage involves cough identification, where algorithms are designed to detect and isolate cough instances within the audio signals. The second stage takes the identified cough segments and performs classification to assign them to the corresponding respiratory disease. Such approaches are presented in [11,12,14]. A notable example is [32] where the YaMNet model is employed to identify the cough segments within the audio signals. Subsequently, a set of time-frequency features are extracted from these cough segments and used as inputs to ML models for the final classification of the cough sounds, enabling the identification of the underlying respiratory disease. Finally, certain works deviate from the aforementioned framework and adopt alternative approaches such as Hidden Markov Models (HMMs) [22].

## 4. Lung and Breath Sounds Analysis of Lower Respiratory Symptoms

The same structure of review analysis is also followed in this Section, which outlines, in detail, our findings regarding the studies that are related to using lung sounds to analyze symptoms, which correspond to lower respiratory diseases, to properly and automatically recognize them by following the AI principles, as well as to identify the disease they are correlated with. Table 2 presents a summary of the studies that are presented in this Section.

### 4.1. Data Acquisition

The identification of respiratory sounds or/and diseases is heavily connected with the device utilized for the data acquisition procedure. The employed device should be able to capture adventitious sounds, which are prominent in low frequencies. Moreover, the data acquisition device should be as sensitive as possible, since the thorax and the larynx act like a low-pass filter for different lung sounds, thus minimizing the audio signal’s amplitude and making it harder to capture.

**Figure 9:**
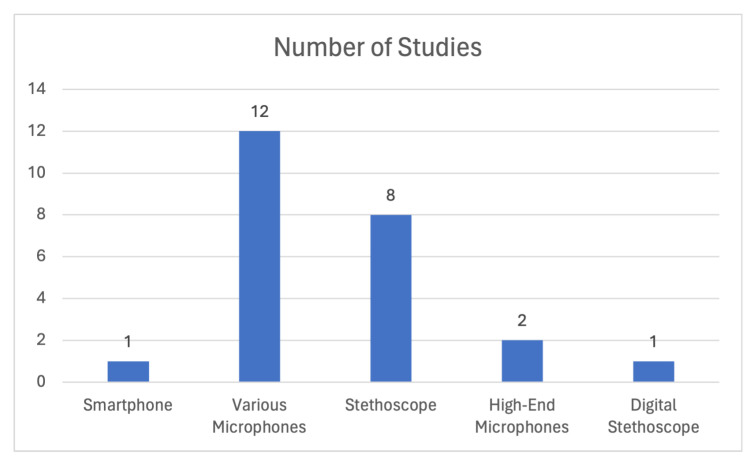
Distribution of respiratory sounds related studies with respect to the device used for data acquisition.

Another important aspect of the data collection stage is the placement of the recording device on the patient’s body and the type of the device. The most common approach is placing the device on the chest area, like a typical auscultation device. This type of data acquisition can be conducted by digital stethoscopes, and the authors in [47,52,53,56,58,59,60,61,62,63,66] utilized data which were derived from this kind of data retrieval methodology. Most of the studies mention that they did not execute the data acquisition phase themselves, but they exploited open-source or proprietary datasets. However, there are also a few studies that did construct their own datasets. It is also common to combine digital stethoscopes and high-impedance microphones as the devices employed for the data collection, in order to provide variability in the quality of the recordings. This results in an increase in the models’ generalization capability, while also expanding the field of application where the trained algorithm can be used. Data constructed by this methodology are employed in [44,51,54,55,57,61,62,67,68]. On the other hand, only a limited number of studies have employed smartphone recordings as a means of data collection, with one notable example being [63], which utilized various smartphones to record lung sounds. Some studies, such as [49], created a custom device in order to capture the recordings. Such devices are external microphones capable of connecting with smartphones. Figure 9 presents a categorical analysis of the devices used for data acquisition in the aforementioned studies. The figure demonstrates that the majority of studies utilized stethoscopes, while high-end microphones and smartphone microphones were less frequently used for data collection in the reviewed literature.

It is worth noting that, as observed in recent studies, recording the patient’s breath can also yield promising results for identifying respiratory sounds. Typical methods for acquiring breath recordings are either by smartphone or high-quality microphone. The authors of [43,48,49] used breath sound recordings deriving from such devices in order to classify lung sounds or diseases.

The procedure of data collection foreshadows the application and utilization of the developed algorithm or model. Until now there is a limited number of available datasets that can be exploited for the identification of respiratory diseases and respiratory sounds. Specifically, there are three available datasets, and they are used by most of the community for research in the domain of respiratory diagnosis. These datasets are the Respiratory Sound Database dataset [69], the Coswara dataset [39], and the R.A.L.E [70] dataset.

The Respiratory Sound Database, in particular, was created by two research teams in Portugal and Greece. It includes 5.5 h of data with 920 annotated recordings. These recordings were acquired from 126 patients. Regarding the respiratory sounds, it contains 1864 crackles, 886 wheezes, and 506 both crackles and wheezes. The data include both clean respiratory sounds as well as noisy recordings that simulate real-life conditions. The patients span all age groups—children, adults, and the elderly. The devices used for the data collection are both a digital stethoscope and a microphone.

The Coswara dataset is based on the Coswara project by the Indian Institute of Science (IISc), Bangalore, in an attempt to build a diagnostic tool for COVID-19 detection using audio recordings, such as the breathing, coughing, and speech sounds of an individual. The data collection stage was conducted through crowdsourcing and the devices used are either smartphones or webcam microphones.

Finally, the R.A.L.E operates as a computer-aided instruction program on chest auscultation originated at the Respiratory Acoustics Laboratory (Prof. H. Pasterkamp, MD, FRCPC), Dept. of Pediatrics and Child Health, University of Manitoba, in Winnipeg, Canada. This dataset was created for teaching purposes, but there are several studies that utilize it. The data in this dataset were acquired by digital stethoscopes. It is important to mention that this dataset is not open-source but it can be purchased.

### 4.2. Domain Focus of Sound Analysis Studies for the Lower Respiratory Symptoms

The main goal of studies related to respiratory diagnosis using artificial intelligence may be categorized into two major branches: (a) disease classification, and (b) the classification of the respiratory sound (crackles, wheezes, etc.).

**Figure 10:**
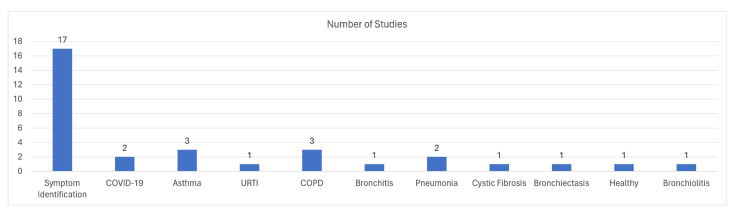
Distribution of studies with respect to their research topic in the area of respiratory sounds.

Regarding the identification of diseases, the focal points are COPD, asthma, COVID-19, bronchitis, Lower Respiratory Tract Infections (LRTI), Upper Respiratory Tract Infections (URTI), and pneumonia. Due to the existing limitations on dataset availability and the difficulties in creating new datasets, studies that cover most diseases are rare. However, it has been observed that research conducted in this domain may focus on multiple diseases at the same time if they have similar attributes. Specifically, the authors of [43,45] focus on asthma detection through the identification of wheezes and achieve an accuracy of 75.21% and 88%, respectively, whereas [47] includes asthma alongside other diseases for disease classification and achieve an accuracy of 60% and 95.67%. One of the most studied diseases for identification or classification is COPD, and [46,47] introduced ML algorithms for COPD identification, while [46] developed a methodology that reached 100% accuracy. Moreover, with the rise of COVID-19, more and more research groups (see e.g., [48,49]) have focused on the diagnosis of COVID-19 from breathing recordings from a smartphone device. The pipeline followed in [49] achieved 69% regarding the precision metric and 0.8 with respect to the AUC metric, while [48] achieved a 100% classification accuracy. Other diseases have also been studied in [44], such as pneumonia, bronchitis, LRTI, and URTI, achieving an identification accuracy of up to 99%. Nevertheless, most studies are focused on respiratory sounds, since they can be correlated with almost all diseases or at least provide useful information for a preliminary diagnosis. The studies [47,51,52,53,54,55,56,57,58,59,60,61,62,64,65] provide different techniques and methodologies for the identification of respiratory sounds, like crackles and wheezes, with promising results. The accuracy achieved in these studies is between 73% and 98%. Figure 10 provides an overview of the distribution of studies with respect to the respective research topics for each study. The literature primarily focuses on the identification of respiratory sounds and symptoms, while respiratory disease identification receives relatively less attention. Among respiratory diseases, asthma, and COPD are the most studied subjects in the reviewed literature.

### 4.3. Implementation Approach

The techniques employed in each study depend on the objective and the data used. The data engineering methodologies fall into two major categories, namely feature-based processing, and transformation-based processing. The first one corresponds to Feature Extraction, and the output is subsequently fed into an ML algorithm for training, whereas the second one corresponds to transforming the raw audio data, using filtering, Mel spectrograms, and more. One important aspect of the methodology is the learning algorithms employed, which can be divided into statistical learning models and Artificial Neural Networks.

Delving into the data processing field that is followed for implementation, the studies [43,45,46,47,48,49,51,54,55,60,61,62] perform Feature Extraction on the data; however, the Feature Extraction comes after several preprocessing steps, namely filtering, upsampling or downsampling, or other transformations. The studies [45,46,49] extract several statistical and spectral features, such as spectral bandwidth, zero-crossing rate, spectral roll-off, etc. ML algorithms that exploit different statistical and spectral features can see a significant improvement in their prediction capabilities by utilizing feature selection techniques. Additionally, the authors in [47] compute features from the power spectral density of the audio data, and the Analysis of Variance (ANOVA) feature selection method is utilized, so only the most important features are utilized in the training phase. Moreover, in [48], both statistical features and feature maps, extracted from a Convolutional Bidirectional LSTM Network, are combined in order to provide more descriptive features of the audio data. Some interesting approaches are utilized by [54,55]. The first study proposed a method of feature-band attention by analyzing the fluid-solid coupling simulation of a bronchial model. As an alternative, the second study performs multiple preprocessing steps in order to extract features; the processing pipeline is constructed by downsampling the audio data, slicing it to windows, filtering, and performing decomposition using the Empirical Mode Decomposition (EMD) method. Afterward, features are extracted to train the algorithm. Lastly, the authors of [60] exploited spectral features originating from the decomposed signal after performing the Empirical Mode Decomposition technique.

As mentioned above, data engineering consists of feature-based methodologies and transformation-based methodologies. The studies [44,47,52,53,56,57,58,59,64,65] follow various transformation methodologies, in order to convert the audio signal to a more manageable form, surfacing as much helpful information as possible. It is a common procedure to transform vector-shaped data into an image-like form by performing time–frequency domain analysis. The authors of [58] converted the lung sound data to Mel spectrograms. In Ref. [64], the researchers tested various ways of representing lung sounds over time and frequency to create spectrogram features which were combined with different spectral, melodic, and MFCCs features to identify respiratory disease symptoms. Another relevant study [65], transformed respiratory cycles from auscultation recordings into Short-Time Fourier Transform (STFT) spectrograms. Moreover, a common approach is to perform a Continuous Wavelet Transform (CWT), as [47,52] proposed, by using a mother wavelet to extract the desired signal. In addition, the authors of [52] applied additional processing methodologies after the transformation, such as the application of a Gaussian filter and Stacked Autoencoder, in order to filter and alternate the representation of the input signal. An alternative way to represent an audio signal is by extracting the MFCCs instead of the Mel spectrogram.

The studies [44,59] utilize this type of conversion. The first study extracts the MFCCs after performing data augmentation, in order to provide variability to the training data. The second study employs a combination of the MFCCs with the coefficients of an autoregressive model (AR model) as the training data. The latter one, prior to the MFCCs calculation, decomposes the signal utilizing the EMD technique, which is a preferred methodology for the decomposition of respiratory sound recordings. A remarkable data processing methodology is noted in [53], in which the authors proposed a novel transformation by optimizing the *S*-transform technique for processes regarding respiratory sounds, especially crackles and wheezes audio recordings. The optimization provides a more detailed depiction of the frequency ranges in which the sounds of interest operate. Lastly [57], recommend an approach based on spectrogram extraction after applying various normalization methods, such as Root Mean Square (RMS) normalization [50], peak normalization, and loudness normalization.

It is widely known that deep neural networks require a large amount of data to achieve good generalization, yet usable data are limited in this field. Consequently, researchers have turned to data augmentation techniques to generate additional usable artificial data from existing instances, thereby improving model performance [57,62]. Notably [62] employs standard augmentation techniques, such as noise addition, speed variation, random shifting, and pitch shift, as well as a novel concatenation-based augmentation technique. This technique involves randomly sampling two samples of the same class and concatenating them to create a new sample for that class.

The other most important aspect of the implementation methodology is the ML algorithm utilized. Some studies employ statistical models, especially those using feature-based data processing. Other studies use ANNs or state-of-the-art neural network topologies. The studies [43,46,47,51,52,56] use SVM and variations of SVM as the model of choice. Alternatively, the authors of [46] utilize fine Gaussian SVM. Moreover, it is a common technique to utilize the available data by training more than one model in order to provide perspective on how the data behaves with different models. The authors in [51] also train an ANN, and both [51] and [56] train a Random Forest Classifier (RF), whereas [47] employ a Neural Network, and unsupervised learning by using the *K*-Nearest Neighbors (*K*-NN) algorithm. Refs. [45,55] also exploit the RF model, and [44,56] utilizes the *K*-NN algorithm too. In Ref. [64], classifiers such as Linear Discriminant Analysis (LDA), Support Vector Machine with Radial Basis Function (SVMrbf), Random Undersampling Boosted Trees (RUSBoost), and Convolutional Neural Networks (CNNs) were tested and used to accurately identify respiratory disease symptoms in lung sound recordings. The study also investigated the impact of using different durations of lung sound segments, revealing that the classifiers might implicitly learn to recognize the durations of events. Furthermore, the study [45] proposes a Neural Network in order to detect asthma through extracted features. Other studies that employ ANNs with features as inputs to perform the training procedure are [47,48,60]. The studies [49,53,54,58,65] use some of the state-of-the-art models. In Ref. [65], a hybrid model combining CNN-LSTM architectures was used to classify lung disease symptoms from lung sound recordings. The CNN module extracted crucial features used by the LSTM module to classify symptoms of lung diseases, achieving a maximum classification accuracy of 76.39%. The studies [53,54] use the ResNet model architecture, whereas [49,58] use the VGG-16, VGGish, and VGGish-BiGRU topologies, respectively. The last study, particularly, utilizes a custom GRU-based NN architecture. Finally, a Gaussian mixture model and a custom 2D Neural Network are proposed in the studies [57,59], correspondingly.

## 5. Voice/Speech-Based Analysis for Respiratory Diseases Identification

Adopting the procedure that was followed in Section 3 and Section 4 for our review, in this Section, the third category of sound analysis is conducted, which is related to the human voice or speech sounds and their correlation with respiratory diseases’ symptoms. Again, the table, in this case Table 3 summarizes the studies that were included.

### 5.1. Data Acquisition

One key aspect that holds significant importance in research involving sound datasets, specifically in the context of the human voice, is the process of data acquisition. The quality of the device employed for data collection, particularly the microphone, can considerably impact the properties of the acquired data. In fact, different types of microphones, such as professional-grade or mobile device-compatible microphones, can yield varying results in terms of the captured sound. Moreover, certain devices may exhibit a bias towards specific sounds while suppressing others. For instance, smartphone microphones are often engineered to disregard ambient noise and prioritize the preservation of human voices.

**Figure 11:**
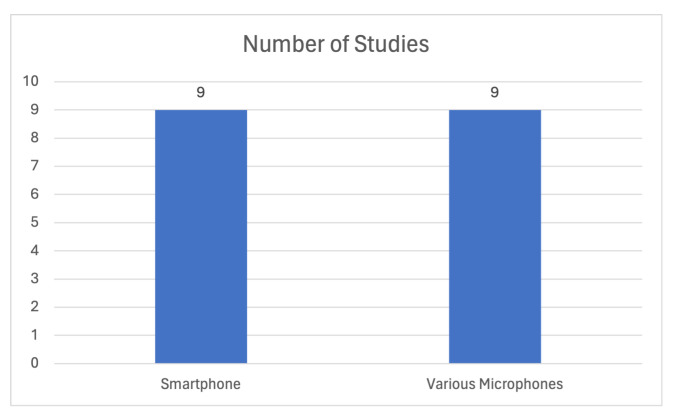
Distribution of studies with respect to the device used for data acquisition.

Regarding the microphone devices that were employed in the data collection process and the creation of the proper datasets, smartphones’ microphones were used in refs. [13,76,78,83,86], plug-in microphones in [71], specialized microphones in [75,82] (one of the specialized microphones used is the ZOOM H6 recorder which records up to a 24-bit depth and 96 kHz), while in other studies, either the source was not specified, or existing benchmark datasets were used. Concerning the latter case, the Coswara dataset was exploited in [72,73,77,87], Crowdsourced Respiratory Sound in [79,87], and Corpus Gesproken Nederlands (*The Corpus Gesproken Netherlands dataset, published in March 2004, was the result of the Corpus Gesproken Netherlands project, which started in 1998 and ended in 2004. The language used is modern Dutch, as spoken by adult speakers in the Netherlands and Flanders. The preparation of this project took place in Ghent and Nijmegen, with the aim of creating a 1000-h spoken corpus. Some recordings were made in-house while others were made in collaboration with external partners.*) in [75]. Figure 11 illustrates the number of studies per device used for data acquisition. Smartphone microphone-related studies are more prevalent compared to those using high-end microphones and specialized recording devices.

Finally, it is worth noting that the sound recorded by microphones is directly affected by the relative position of the microphone with respect to the sound source, which in the case of voice/speech-based respiratory diseases classification, is the mouth of the patient. For that purpose, it is important in all experiments that the microphone is consistently placed in the same spot, as for example in [78,86], where the recording devices were smartphones which were placed on a table 20 cm from each patient’s mouth.

### 5.2. Domain Focus of Voice/Speech Sound Analysis Studies

The primary objective of relevant studies in the existing literature is to classify respiratory diseases using various voice recordings. Typically, these studies concentrate on the classification of a single disease, implying a binary classification scenario.

Consequently, the studies can be categorized based on two criteria: (a) the specific disease under research, and (b) the type of voice recording data utilized. In relation to disease identification, for COVID-19, several studies [13,71,72,73,74,77,78,87] have been conducted with accuracy rates ranging from 73% to 97% [73]. Similarly, studies have also focused on asthma [80,81,82,83,84], achieving accuracy scores ranging from 74% to 100% [80], and COPD [75], with an accuracy of 75% [76]. Notably, one study [76] suggests the potential to achieve a Matthews Correlation Coefficient of 1, indicating a correct classification rate of 100%.

Regarding the type of voice recording data, studies have utilized vowel sounds such as /a/, /e/, and /o/ [72,73,82], speech recordings including counting numbers and reading sentences [13,75,76,83,84,85], and in some cases, cough recordings [78,79]. Notably, several studies [71,74,77,80,81,86,87] have utilized multiple types of voice recording data.

**Figure 12:**
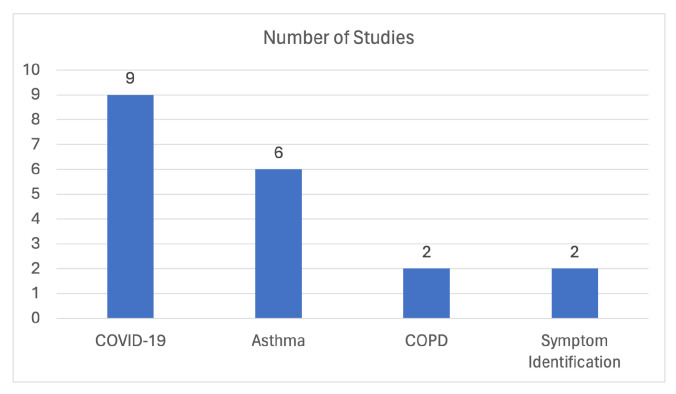
Distribution of studies with respect to their research topic in the area of voice analysis for disease identification.

It is evident that a significant focus has been placed on COVID-19, which is understandable given the recent pandemic, as well as on asthma, which is a prevalent respiratory disease. Figure 12 visually illustrates the distribution of studies based on their respective research topics, and we can observe that most of the research literature emphasizes COVID-19, followed by asthma, and then COPD as the top three primary research topics within this domain.

### 5.3. Implementation Approach

The selection of preprocessing techniques and models employed varies across different studies. The categorization of relevant studies can be based on the type of implemented model, classifying them into either the neural networks category, such as CNNs and Recurrent Neural Networks (RNNs), or the statistical learning-related category, such as SVM and Logistic Regression.

In terms of the data processing methodology, a common practice in the signal processing domain involves statistical audio Feature Extraction, which means generating features from the signal data. In a specific study [79], a Data De-noising Auto Encoder (DDAE) was utilized to extract in-depth acoustic sound signal features. In several studies [13,71,72,73,77,78,79,80,84,85], researchers have employed various computational techniques to compute significant features for analyzing audio signals related to COVID-19 and other applications. These features include MFCCs, which capture the spectral characteristics of the sound, shimmer (measuring amplitude variability), jitter (measuring temporal variability), Spectral centroid (indicating the “brightness” of the audio signal), and others. For instance, in study [71], voice samples consisting of 5 s “ah” sounds, a Thai polysyllabic sentence, and cough sounds were divided into 100 ms sub-samples. The log-mel spectrogram was computed for each sub-sample, resulting in a 2D representation. To make it more suitable for downstream learning, the 2D subsamples were subsequently converted into a 3D representation. In Ref. [72], the authors extracted features such as fundamental frequency, shimmer, jitter, harmonic-to-noise ratio, MFCCs, spectral centroid, and spectral roll-off. These features were then used as inputs to their respective models. Moreover, in the DiCOVA challenge [77], the authors extracted 3D MFCCs features along with the Δ and ΔΔ coefficients. They used a window size of 1024 samples and a hop size of 441 samples. Additionally [78], employed a two-step preprocessing approach. First, they generated the mel-spectrum representation from the recordings using 25 ms frames and 10 ms overlaps. Each frame consisted of 80 mel-scaled frequencies and their corresponding 80 first derivatives. Subsequently, a transformer model based on the Mockingjay system [88] was applied. The authors of [79] studied five data augmentation methods, including time-stretching, pitch-shifting, compression-of-range, and the addition of background noise, to enhance the audio data. In another study [80], the authors exploited the Improved Weed Optimization (IWO) algorithm for Feature Extraction and the Enhanced Hunting Search algorithm for signal selection. Asthma prediction [84] involved producing the feature vector through the Ripplet-2 transform, which likely captured relevant information for the task. Lastly, in [85] multiple features were extracted, including formant frequencies, intensity, pitch, shimmer, jitter, mean harmonics-to-noise ratio (HNR), mean autocorrelation, mean noise-to-harmonics ratio (NHR), mean power, mean amplitude, total energy, standard deviation, MFCCs, and linear prediction coefficients. These various Feature Extraction techniques aimed to capture different aspects of the audio signals, providing valuable inputs for subsequent analysis and modeling in the respective studies.

Alternatively, several researchers employ more sophisticated features extracted from specialized software like openSMILE [42], PRAAT [89], and the Computational Paralinguistics Challenge (ComParE) [90] datasets from Interspeech 2010, 2013, and 2016. In particular, openSMILE is extensively employed for audio and speech processing, enabling the generation of a wide array of features, including low-level descriptors (e.g., spectral and prosodic features), higher-level descriptors (e.g., emotion and speaker characteristics), and audio events (e.g., music, laughter, and speech). PRAAT on the other hand, is an open-source software, which is employed for speech analysis, synthesis, and manipulation. Moreover, the ComParE software competition focused on computational approaches to paralinguistic tasks, which involve non-verbal aspects of communication, such as vocal effects, prosody, and intonation. Specifically, the studies [74,75,76,82,83,86] utilize the aforementioned software tools and methods of computational approaches (openSMILE, PRAAT, and ComParE). The number of features extracted and used with the employment of this software can reach the order of thousands. For example, openSMILE includes features based on emotion and intonation beyond common statistical features. In Refs. [75,82,86] 6373 features were extracted through ComPare2016 and ComPare2013, respectively, in [76] 103 features were extracted through PRAAT and openSMILE, and in [83] 1582 features in total were extracted and then normalized with the standard mean and variance normalization. Moreover, in [74] a data cleansing phase took place, where voice recordings shorter than 500 ms and cough recordings shorter than 100 ms were discarded while 5% of the /a/ vowel sounds were trimmed to avoid inhale and exhale effects on the recording. Additionally, three sets of features were extracted, 6373 features from the ComParE software, 65 features from PRAAT and Librosa [91], and a 1024 embedding feature vector from a D-CNN (Deep CNN) model which was trained on the ESC-50 dataset. Lastly, in [13], the researchers processed the data samples using the PRAAT software. Specifically, they extracted features such as spectral centroid and roll-off, zero-crossing rate, MFCCs, and the first and second-order time derivatives of MFCCs.

Regarding the types of models utilized for voice analysis in the cited studies, we can observe two distinct groups of methodologies. The first group focuses on employing neural networks for the classification of respiratory diseases, while the second group relies on ML models that are related to statistical learning theory, such as SVM and RF.

In the studies [13,71,79,84], the first group utilizes neural networks for voice analysis. Specifically, in [71] the researchers employed a pre-trained state-of-the-art CNN known as VGG-19. Subsequently, transfer learning techniques were applied to adapt the model’s weights to the corresponding audio dataset. Notably, the model’s architecture was slightly modified, wherein the output layer was replaced with a new one featuring a sigmoid activation function and a single unit. Additionally, two dense layers, each containing 64 and 32 fully connected units, respectively, were introduced. The Adam optimizer was utilized for training, and the model’s performance was evaluated using the 3-fold cross-validation technique. In a similar vein [79], also employed a 1D-CNN architecture while incorporating a data de-noising auto encoder (DDAE) to facilitate automated feature learning. In particular, the model consists of six 1D CNN layers, average pooling layers, and fully connected layers with the ReLU activation function, while in the last layer, the Softmax activation function is used for classification. In Ref. [13], the researchers have developed a hybrid model that combines the architectural elements of a Long Short-Term Memory (LSTM/RNN) model. The LSTM component comprises 512 LSTM units and is augmented with a dropout layer featuring a dropout rate of 0.5, a dense layer, and a Flatten layer. During training, the Adam optimizer was employed to optimize the model’s parameters, and the Rectified Linear Unit (ReLU) activation function was used to introduce non-linearity to the model. In the context of [84], accordingly, the authors have utilized a Recurrent Deep Neural Network (RDNN) as a classifier. The RDNN architecture is composed of a Don-Sigmoid layer and a Record-Sigmoid layer, which are essential components for its functioning as a classifier in the given context.

Conversely, the studies [72,73,74,75,76,80,82,83,85,86,87] fall into the second group, employing models that are based on the statistical learning theory, like SVM and RF, for voice analysis. It is worth highlighting that SVM is the most commonly used ML model for respiratory disease classification due to its effectiveness on small and complex datasets. However, researchers also frequently utilize the Random Forest and *K*-Nearest Neighbors algorithms for this purpose.

## 6. Publicly Available Datasets

Since the COVID-19 pandemic erupted, many scientific research groups have been involved in providing state-of-the-art respiratory disease detection solutions. Due to this, several datasets have emerged to help researchers design and evaluate their innovative solutions experimentally. These datasets focus on respiratory disease classification, cough detection, respiratory sounds classification, or a combination of them. This section, in particular, contains the datasets that are publicly available, and which could be utilized by the community without license concerns. Moreover, because of the nature of the research in this section, it is worth mentioning that we excluded our policy for filtering the studies regarding our systematic review. Table 4 presents an overview of the datasets that were analyzed in this study.

To begin with, the *COUGHVID* [92] dataset is an open-source dataset, which includes 25,000 crowdsourced instances of coughs obtained from both COVID-19-infected and healthy patients. Another dataset is the COVID-19 Sounds [38] dataset, developed by the University of Cambridge, which includes recordings of coughs as well as breath and speech samples. Additionally, the *Coswara* [39] and the Respiratory Sound Database [69] datasets are among the most prominent resources. In particular, the *Coswara* is a crowdsourced dataset originating from the Indian Institute of Science, specifically designed for COVID-19 research. It contains instances of coughs, speech, and breathing from 2747 users, making it suitable for various COVID-19 detection applications. Respectively, the Respiratory Sound Database dataset has gained popularity as it was utilized in the 2017 ICBHI Challenge. It consists of auscultation recordings that were retrieved using three digital stethoscopes and a high-quality microphone, involving 920 patients. The audio instances are annotated for a number of diseases, such as asthma, COPD, LRTI, and URTI. Notably, each respiratory cycle within the recordings is also annotated based on the existence of crackles, wheezes, both crackles and wheezes, or the absence of these adventitious sounds, making it ideal for disease or lower respiratory disease symptom sound classification tasks. Another dataset for respiratory sound classification is the SPRSound dataset [95], which was created within the context of IEEE BioCAS 2022 Respiratory challenge [99]. It consists of 2683 records and 9089 respiratory sound events from 292 participants. The authors employed 11 experienced paediatric physicians to annotate it, using a custom-made software. “HF_Lung” [96] database stands as the last identified database for lung sound analysis. This database originally had lung sounds recorded from 18 patients between August 2018 and October 2019. Then, in 2020 the database was updated with data acquired from 261 patients.

Other datasets that focus on cough instances are the *Corp* [40], which comprises cough recordings from patients with various respiratory diseases, and the *Virufy* dataset [37], which combines cough recordings from the *COUGHVID* and *Coswara*’s cough recordings. Furthermore, the Tos-COVID-19 [94] should be mentioned since it contains cough recordings; however, it should be noted that the metadata in this dataset is in Spanish. In terms of datasets focused on respiratory sounds the *COVID-19 + Pulmonary Abnormalities* [93] dataset stands out, as it is the only dataset containing images, specifically spectrograms, from COVID-19 patients. It is important to mention that most of the images are generated and not extracted from real patient recordings. Finally, the ESC-50 [97] and the Audioset [98] large-scale datasets deserve a mention, as although their primary focus does not align with the subject covered in this systematic review (even though they contain a number of instances related to respiratory disease symptoms recordings), they can provide valuable data and recordings for evaluating the robustness of AI models. Moreover, they can be exploited to pre-train the neural network models in order to understand the formation of sound recordings, and then, post-train these models with the data that correspond to the actual problem.

## 7. Discussion

In this review, a total of seventy-five (75) studies focusing on the utilization of audio biomarkers for respiratory symptom and disease identification were shortlisted and categorized into three domains with respect to the chosen audio biomarker: cough sounds, lower respiratory symptom (lung/breath) sounds, and voice/speech sounds, chosen for their research purposes. It is worth noting that, although the number of studies related to the cough audio biomarker is larger compared to studies related to the other two domains, the selected studies collectively represent the latest state-of-the-art trends across all three domains. In summary, the most relevant studies were chosen for each domain, resulting in an equal distribution of studies per topic. To the best of our knowledge, the included studies depict the state-of-the-art in this domain.

A remarkable note is the significant rise in the number of studies regarding the identification and diagnosis of COVID-19, which seems to align temporally with the outburst of the pandemic. A large proportion of the included studies were published during the duration of the pandemic, mainly in 2020, 2021, and 2022, and are dedicated to this topic instead of focusing on a broader range of respiratory diseases. In particular, COVID-19 affected all three focus areas of this review; among the 33 studies examining cough sounds, 15 focus their research on COVID-19, whereas; among the 24 on respiratory sounds classification and symptom identification, 2 studies specifically address COVID-19. Furthermore, out of the 18 studies on voice/speech analysis, 9 focus on COVID-19. By combining all three focus areas, these studies constitute approximately one-third of the total presented.

It is observed that 17 out of the 24 studies examining respiratory sounds primarily focus on identifying symptoms such as wheezes and crackles, without further examining the underlying diseases. Additionally, as far as cough-related studies are concerned, one-third of them mainly focus on recognizing and identifying cough sounds instead of disease identification. Finally, regarding voice/speech analysis, most of the reviewed literature focuses on disease identification, with the majority of the studies emphasizing COVID-19.

### 7.1. Challenges

Elaborating on the aforementioned observations, the current state-of-the-art is capable of extracting meaningful outcomes through the analysis of audio biomarkers regarding the successful identification of an existing respiratory disease or creating dependable patient health monitoring systems. In terms of evaluation, most studies present notable results and validate the basis of their approaches. Even though there is a large number of relevant studies, we only identified a few studies that are close to delivering or have already delivered a final product. This could be attributed to several factors such as the difficulty in acquiring trustworthy and real-world representing data and data labeling. Thus, these tasks, which are highly dependent on the data show that it is difficult to create production-ready software that can be released for public usage.

### 7.2. Opportunities

Despite the challenges, many different opportunities arise in the domain of respiratory disease diagnosis using audio sounds and artificial intelligence. For instance, high-end devices used to identify intricate respiratory sounds are starting to be accommodated by smartphones as well. In our view, a breakthrough could emerge from the integration of new technologies and methodologies. The constant evolution of mobile devices with respect to processing power and the constant improvement of the modules they integrate (e.g., better, more sensitive microphones) can enable a more reliable data collection procedure and non-invasive health monitoring. Additionally, remote diagnosis systems could be incorporated into such devices, especially through the advancements occurring in the field of edge computation. An important observation is that studies until now explore one audio biomarker. The analysis of more than one sound, simultaneously, may result in improved (but more complex) systems and models that can provide a more complete representation of the patient’s health status and the monitoring of disease severity, or even act as reliable diagnosis-assistive tools for physicians. Additionally, remarkable research could take place by combining respiratory sound features with other vital signals, bio-parameters, or bio-imaging features, which could improve the automatic smart diagnosis both for upper and lower respiratory abnormalities, even in a non-invasive manner. For instance, such biomarker data could be retrieved from smart IoT (Internet of Things) devices.

Moreover, it is worth noting that the research on collecting more reliable data related to respiratory symptoms is significant, as the AI problems are data-driven, which can yield better, robust, and trustworthy solutions regarding the identification of the relevant diseases or symptoms. Even though our research took place until the 30th of November 2022, several efforts have arisen lately regarding the creation of benchmark datasets. For instance, the authors of [100] created a multi-source database that includes respiratory sounds from ICU COVID-19 patients, along with X-rays, heart sounds, LUS, and ICU parameters.

Finally, AI and audio analysis could be promising for the automatic identification of comorbidities, which are a significant topic that should be discussed as a short- and long-term need. Such issues are related to the topic of neurodegenerative diseases linked with voice features or in automatic intelligent analysis of Premature Atrial Contractions (PACs) where comorbidities are a key factor.

## 8. Conclusions

It is nearly impossible to find an individual who has not encountered a respiratory disease at some point in their life. Traditionally, the assessment of respiratory diseases involves the expertise of medical professionals skilled in the on-site examination of the respiratory system through the analysis of respiratory sounds, such as coughing and upper and lower respiratory tract sounds, as well as vocalizations. However, recent advancements in the field of ML significantly contributed to the proliferation of research in automating the diagnosis of these diseases by leveraging the aforementioned respiratory sounds. In this study, we presented how these advancements in technology enable the intelligent analysis of respiratory disease.

This comprehensive review, specifically, examined seventy-five (75) studies, categorizing them into three domains: cough sounds, lower respiratory symptom (lung/breath) sounds, and voice/speech sounds. Although the majority of studies focused on cough sounds, the selected studies collectively represented the latest state-of-the-art trends across all three domains. A notable observation was the significant rise in studies dedicated to identifying and diagnosing COVID-19, coinciding with the pandemic outbreak. This surge impacted all domains, with a substantial number of publications focused on COVID-19 during this period. Regarding respiratory symptoms’ detection, while it predominantly relies on specialized external digital stethoscope devices, there’s a burgeoning trend and notable promise in developing mobile apps for similar purposes.

This rapidly advancing domain shows significant potential in transforming healthcare practices, both in institutional settings and home environments. Finally, it is worth noting that the advancements in edge computing, hardware, and multi-biomarker analysis also offer promising avenues for transcending current limitations and revolutionizing respiratory healthcare, enabling a more comprehensive understanding of a patient’s health status and providing valuable diagnostic tools for medical practitioners.

## Figures and Tables

**Table 1:** Cough sounds analysis related papers.

Reference	Year	Topic	Data and Cohort	Recording Device	ML Models Used	Data Processing Methods	KPIs
[8]	2020	Cough Detection	Private-110 subjects (various resp. diseases)	Various Microphones	XGBoost, RF, DT	Feature Extraction from cough events	Acc: 93% Sens: 97% Spec: 95
[9]	2020	Disease Identification: COVID-19	Private-2660 subjects	Web App, Various Microphones	CNN w/1 Poisson biomarker and 3 pre-trained ResNet50	MFCCs	AUC: 97%
[10]	2020	Disease Identification: COVID-19	ESC-50, Audioset-Cohort n.s.	Various Microphones	CNN, VGG16	MFCCs	Accuracies: COVID/Non-COVID: 70% Cough/Non-Cough: 90%
[11]	2021	Disease Identification: COVID-19	Private-8380 positive, 6041 negative instances	N/A	2D CNN	EMD and MFCCs	AUC: 97%
[12]	2021	Disease Identification: COVID-19	Coswara, Virufy-Cohort n.s.	Web App, Various Microphones	SVM, KVM, RF	Common Short Term Features and MFCCs	AUC: 90%
[13]	2021	Disease Identification: COVID-19	Private-240 acoustic data—60 normal, 20 COVID-19 subjects	Smartphone	LSTM (RNN)	Spec. Centroid, Spec. roll-off, ZCS, MFCC (+ΔΔ)	Acc: 97%, F1: 97.9%
[6]	2021	Disease Identification: COVID-19	Private-Cohort n.s.	Crowdsourced audio recordings, Various Microphones	XGBoost	Feature Extraction	Acc: 97%
[14]	2020	Cough Detection, Disease Identification: COVID-19	ESC-50 and Private-543 Recordings (96 bronchitis, 130 pertussis, 70 COVID-19, 247 Normal)	N/A	1 CNN for Cough Classification and 3 CNNs for COVID-19 detection	Mel-spectrograms to images	Accuracies: Cough Detection: 95.5% COVID-19 Identification: 92.64%
[15]	2021	Disease Identification: Bronchitis, Asthma, COVID-19, Healthy	N/A	N/A	Fully connected NN layer	Questionnaire and Cough Embeddings	Acc: 95.04%
[16]	2022	Cough Detection, Disease Identification: COVID-19	Virufy-Cohort n.s.	N/A	DNN	Windowing and Feature Extraction	Acc: 97.5%
[17]	2022	Cough Detection	Corp Dataset-42 volunteers (18 CAP, 4 asthma, 17 COPD, 3 other resp. illness)	Digital Recorder	CNN	MFCCs	Acc: 99.64%, IoU: 0.89
[18]	2022	Cough Detection	Public/ Private-3228 cough and 480,780 non-cough sounds	Various Microphones	GB Classifier	Feature Extraction and manual selection	Acc: 99.6% (Validation only on hospital data from children)
[19]	2022	Cough Detection, Disease Identification: COVID-19	Cambridge, Coswara, Virufy, NoCoCoDa - Cohort n.s.	Various Microphones	Adaboost, MLP, XGBoost, Gboost, LR, *K*-NN, HGBoost to MCDM	Feature Extraction	Acc: 85%
[5]	2022	Disease Identification: COPD, AECOPD	Private-177 volunteers (78 COPD, 86 AECOPD, 13 not used)	Various Microphones	N/A	Feature Extraction	ROC Curve: 0.89, agreement w/ clinical study
[20]	2013	Symptom Identification: Wet Cough, Dry Cough	Private-78 Patients	High-end Microphones	LR	Feature Extraction	Sens: 84%, Spec: 76%
[21]	2020	Cough Detection	Private-26 healthy participants	Smartphone	*K*-NN, DT, RF, SVM	Feature Extraction and Selection	F1 Score: 88%
[22]	2019	Cough Detection	Private-20 min of cough sounds	N/A	Hidden Markov Models	Single and Multiple Energy Band	AUC: 0.844
[23]	2020	Cough Detection	Private-94 adults	Smartphone	CNN	Mel spectrograms	Accuracies: Cough Detection: 99.7% Sex Classification: 74.8%
[24]	2018	Cough Detection, Disease Identification: Croup	Private-56 croup and 424 non-croup subjects	Smartphone	SVM and LR	Feature Extraction	Sens 92.31% Spec: 85.29% Croup classification Acc: 86.09%
[25]	2020	Cough Detection	N/A	N/A	SVM and *K*-NN	Feature Extraction	Accuracies: *K*-NN: 94.51%, SVM: 81.22%
[26]	2020	Symptom Identification: Wet Cough, Dry Cough	Private-5971 coughs (5242 dry and 729 wet)	Smartphone	RF	Feature Extraction (Custom and OpenSmile)	Acc: 88%
[27]	2021	Disease Identification: Heart-failure, COVID-19, Healthy	Private-732 patients (241 COVID-19, 244 Heart-failure, 247 Normal.	Smartphone	*K*-NN	DNA Pattern Feature Generator, mRMR Feature Selector	Acc: 99.5%
[28]	2020	Cough Detection, Disease Identification: Pertussis, Bronchitis, Bronchiolitis	ESC-50, Audioset-Cohort n.s.	Various Microphones	CNN	Mel-spectrograms	Accuracies: Disease Identification: 89.60%, Cough Detection: 88.05%
[29]	2019	Cough Detection, Symptom Identification: Productive Cough, Non-productive Cough	Private-810 events: 229 non-productive cough, 74 productive cough, and 507 other sounds	Various Microphones	DLN	FFT and PCA	Acc: 98.45%
[30]	2017	Disease Identification: Croup	Private-364 patients (43 Croup, 321 non-croup	Smartphone	LR and SVM	MFCCs and CIFs	Acc: 98.33%
[4]	2020	Disease Identification: Asthma	Private-997 asthmatic, 1032 healthy sounds	Smartphone	GMM - UBM	MFCCs and CQSSs	Acc: 95.3%
[31]	2021	Disease Identification: COVID-19	Coswara-Cohort n.s.	Smartphone	ResNet50	Feature Extraction	Acc: 97.6%
[32]	2022	Disease Identification: COVID-19	Coswara, Virufy, Cambridge and private-Cohort n.s.	Various Microphones	Most popular supervised models	Feature selection and extraction	Best Acc: Random Forest: 83.67%
[33]	2022	Disease Identification: Tuberculosis	Private-16 TB and 35 non-TB patients	Various Microphones	LR, SVM, *K*-NN, MLP, CNN	Feature Extraction	Best Acc: LR: 84.54%
[34]	2021	Disease Identification: COVID-19	Virufy, NoCoCoDa-Cohort n.s.	Various Microphones	SVM, LDA, *K*-NN	Feature Extraction and SFS feature selection	Best Acc: *K*-NN: 98.33%
[7]	2021	Cough Detection	ESC-50-50 cough recordings	Various Microphones	CNN	Mel spectrograms and Data Augmentation	Acc: 98%
[35]	2021	Disease Identification: COVID-19	Coswara, COVID-19 Sounds-Cohort n.s.	Various Microphones	Contrastive Learning	Contrastive Pre-training: Feature Encoder w/ Random Masking	Acc: 83.74%
[36]	2019	Disease Identification: Asthma, Healthy	Private-89 children for each cohort, 1992 healthy and 1140 asthmatic cough sounds	Smartphone	Gaussian mixture model	Downsampling and multidimensional Feature Extraction (MFCCs and CQCCs)	Sens: 82.81%, Spec: 84.76%, AROC: 0.91

Note. **ML models:** SVM = Support Vector Machine; *K*-NN = *K*-Nearest Neighbors; DT = Decision Trees; RF = Random Forest; NN = Neural Network; CNN = Convolutional Neural Network; RNN = Recurrent Neural Network; DLN = Deep Learning Networks; MLP = Multilayer Perceptron; LSTM = Long Short-Term Memory; GB classifier = Gradient Boosting classifier; HGBoost = Hyperoptimized Gradient Boosting; LR = Linear Regression; GMM = Gaussian Mixture Model; UBM = Universal Background Models; LDA = Linear Discriminant Analysis. **Data Processing Methods:** MFFCs = Mel-Frequency Cepstral Coefficient; mRMR = minimum Redundancy − Maximum Relevance; FFT = Fast Fourier Transform; PCA = Principal Component Analysis; CIF = Cochleagram Image Features; CQCC = Constant-Q Cepstral Coefficients. **Metrics:** Acc = Accuracy; Sens = Sensitivity; Spec = Specificity; AUC = Area Under Curve; IoU = Intersection over Union; ROC Curve = Receiver Operating Characteristic Curve; AROC = Area under the ROC curve.

**Table 2:** Respiratory sounds analysis related papers.

Reference	Year	Topic	Data and Cohort	Recording Device	ML Models Used	Data Processing Methods	KPIs
[43]	2018	Symptom Identification: Wheeze	Private-255 breathing cycles, 50 patients	Smartphone	SVM	Bag-of-Words To Features	Acc: 75.21%
[44]	2020	Disease Identification: Bronchitis, Pneumonia	Private-739 recordings	Various Microphones	*K*-NN	EMD, MFCCs, GTCC	Acc: 99%
[45]	2021	Disease Identification: Bronchial Asthma	Private-952 recordings	High-end Microphones	NN, RF	Spectral Bandwidth, Spectral Centroid, ZCR, Spectral Roll-Off, Chromacity	Sens: 89.3% Spec: 86% Acc: 88% Youden’s Index: 0.753
[46]	2019	Disease Identification: COPD	Private-55 recordings	Stethoscope	Fine Gaussian SVM	Statistical Features, MFCCs	Acc: 100%
[47]	2018	Disease Identification: Asthma, COPD	Private-80 normal, 80 COPD, and 80 asthma recordings	Stethoscope	ANN	PSD Extracted Features, Feature Selection (ANOVA)	Acc: 60% Spec: 54.2%
[48]	2022	Disease Identification: COVID-19	Coswara-120 recordings from COVID-19 patients, 120 recordings from Healthy patients	Various Microphones	Neural Network	Statistical and CNN-BiLSTM Extracted Features	Acc: 100% (shallow recordings), 88.89% (deep recordings)
[49]	2021	Disease Identification: COVID-19	COVID-19 Sounds-141 recordings	High-end Microphones	VGGish	Spectral Centroid, MFCCs, Roll-off Frequency, ZCR	ROC-AUC: 80% Prec: 69% Recall: 69%
[50]	2022	Symptom Identification: Wheeze, Crackle	Respiratory Sounds Database (RSDB) and private-943 recordings	Various Microphones	ResNet	Padding, STFT, Spectrum Correlation, Log-Mel Spectrograms, Normalization	Sens: 76.33% Spec: 78.86%
[51]	2021	Symptom Identification: Wheeze, Crackle	RSDB-920 recordings	Various Microphones	ANN, SVM, RF	Time Statistics and Frequency features	Acc: NN: 73%, RF: 73%, SVM: 78.3%
[52]	2019	Symptom Identification: Wheeze, Crackle	Private-21 normal samples, 12 wheezes, and 35 crackles	Stethoscope	SVM	CWT, Gaussian Filter, Average Power, Stacked Autoencoder	Acc: 86.51%
[53]	2019	Symptom Identification: Wheeze, Crackle	RDSB’s stethoscope recordings-834 recordings	Stethoscope	ResNet	Optimized S-transform	Sens: 96.27% Spec: 100% Acc: 98.79%
[54]	2022	Symptom Identification: Wheeze, Crackle	RDSB-920 recordings	Various Microphones	VGG-16	Fluid-Solid Modeling, Recording Simulation, Downsampling, Feature Extraction	Sens: 28%, Spec: 81%
[55]	2020	Disease Identification: Bronchiectasis, Bronchiolitis, COPD, Pneumonia, URTI, Healthy	RDSB-920 recordings	Various Microphones	RF	Resampling, Windows, Filtering, EMD, Features	Acc: 88%, Prec: 91%, Recall: 87%, Spec: 97%
[47]	2020	Symptom Identification: Wheeze, Crackle	Private-705 lung sounds (240 crackle, 260 rhonchi, and 205 normal)	Stethoscope	SVM, NN, *K*-Nearest Neighbors (*K*-NN)	CWT	Acc: 90.71%, Sens: 91.19%, Spec: 95.20%
[56]	2021	Symptom Identification: Crackle, Normal, Stridor, Wheeze	Private-600 recordings	Stethoscope	SVM, *K*-NN	Filtering, Amplification, Dimensionality Reduction, MFCCs, NLM Filter	Acc: SVM: 92%, *K*-NN: 97%
[57]	2021	Symptom Identification: Wheeze, Crackle	RSDB-920 recordings	Various Microphones	2D CNN	RMS Norm, Peak Norm, EBU Norm, Data Augmentation	Acc: 88%
[58]	2019	Symptom Identification: Wheeze, Crackle	Private-384 recordings	Stethoscope	VGGish-BiGRU	Spectrograms	Acc: 87.41%
[59]	2017	Symptom Identification: Wheeze, Crackle	Private-60 recordings	Stethoscope	Gaussian Mixture Model	MFCCs	Acc: 98.4%
[60]	2017	Symptom Identification: Wheeze, Crackle	Private-recordings containing 11 crackles, 3 wheezes, 4 stridors, 2 squawks, 2 rhonchi, and 29 normal sounds	Digital stethoscope	MLP	EMD, IMF, Spectrum, Feature Extraction	Acc: Crackles 92.16%, Wheeze 95%, Stridor 95.77%, Squawk 99.14%, Normal 88.36%, AVG 94.82%
[61]	2021	Symptom Identification: Wheeze, Crackle	RSDB-920 recordings	Various Microphones	VGG-16	Resampling, Windows, Filtering, Mel spectrogram (Mel, Harmonic, Percussive, Derivative)	Acc: Wheeze 89.00%, Rhonchi 68.00%, Crackles 90.00%
[62]	2020	Symptom Identification: Wheeze, Crackle	RSDB-920 recordings	Various Microphones	ResNet	Resampling, Windows, Filtering, Data Augmentation, Mel-spectrogram, Device Specific Features	80/40 Split 4 class (per device): Spec: 83.3%, Sens: 53.7%, Score: 68.5%
[63]	2021	Symptom Identification: Crackle, Wheeze – Disease Identification: Asthma, Cystic Fibrosis	Private-Recordings from 95 patients	Various Microphones	N/A	N/A	85% agreement (k = 0.35 (95% CI 0.26-0.44)) between conventional and smartphone auscultation Features
[64]	2021	Symptom Identification: Wheeze, Crackle, Other	RSDB-920 recordings	Various Microphones	LDA, SVM with Radial Basis Function (SVMrbf), Random Undersampling Boosted trees (RUSBoost), CNNs.	Spectrogram, Mel-spectrogram, Scalogram, Feature Extraction	Acc: 99.6%
[65]	2022	Symptom Identification: Wheeze, Crackle, Normal	RSDB-920 recordings	Various Microphones	Hybrid CNN-LSTM	Feature Extraction	Sens: 52.78% Spec: 84.26% F1: 68.52% Acc: 76.39%

Note. **ML models:** SVM = Support Vector Machine; *K*-NN = *K*-Nearest Neighbors; RF = Random Forest; ANN = Artificial Neural Network; NN = Neural Network; CNN = Convolutional Neural Network; MLP = Multilayer Perceptron; RUSBoost = Random Undersampling Boosted trees; LSTM = Long Short-Term Memory; LDA = Linear Discriminant Analysis. **Data Processing Methods:** EMD = Empirical Mode Decomposition; MFFC = Mel-Frequency Cepstral Coefficient; GTCC = Gamatone Cepstral Coefficient; ZCR = Zero-Crossing Rate; PSD = Power Spectral Density; STFT = Short Time Fourier Transform; CWT = Continous Wavelet Transform; S-Tranform = Stockwell Transform; NLM = Non-Local Means; RMS = Root Mean Square. **Metrics:** Acc = Accuracy; Sens = Sensitivity; Spec = Specificity AUC = Area Under Curve; IoU = Intersection over Union; ROC Curve = Receiver Operating Characteristic Curve; AROC = Area under the ROC curve.

**Table 3:** Voice analysis related papers.

Reference	Year	Topic	Data and Cohort	Recording Device	ML Models Used	Data Processing Methods	KPIs
[71]	2020	Disease Identification: COVID-19	Private—116 subjects (76 8 weeks post COVID-19, 40 Healthy	Smartphone, Various Microphones	VGG19	Log-mel spectrogram	Acc: 0.85%, Sens: 0.89%, Spec: 0.77%
[72]	2021	Disease Identification: COVID-19	Coswara—166 subjects (83 COVID-19 positive, 83 Healthy)	Various Microphones	NB, Bayes Net, SGD, SVM, *K*-NN, Adaboost algorithm (model combination), DT, OneR, J48, RF, Bagging, Decision table, LWL	Fundamental Frequency (F0), Shimmer, Jitter and Harmonic to Noise Ratio, MFCC or Spectral Centroid or Roll-Off	Best overall results for vowels a, e, o: Random Forest: Acc: 82.35%, Sens: 94.12%, Spec: 70.59%
[73]	2021	Disease Identification: COVID-19	Coswara—1027 subjects (77 COVID-19 positive (54M, 23F), 950 Healthy (721M, 229F))	Various Microphones	SVM, SGD, *K*-NN, LWL, Adaboost and Bagging, OneR, Decision Table, DT, REPTree	ComParE_2016, FF, Jitter and Shimmer, Harmonic to Noise Ratio, MFCCs, MFCC Δ and ΔΔ, Spec. Centroid, Spec. Roll-off	Best overall results for vowels a, e, o: SVM: Acc: 97.07%, F1: 82.35%, Spec: 97.37%
[74]	2021	Disease Identification: COVID-19	Private—196 subjects (69 COVID-19, 130 Healthy)	Mobile App, Web App–Smartphone, Various Microphones	SVM, RBF, RF	1024 embedding feature vector from D-CNN	Best model: RF: Acc: 73%, F1: 81%
[75]	2022	Disease Identification: COPD	Corpus Gesproken Nederlands—Cohort n.s.	Various Microphones	SVM	Mean intensity (db), Mean frequency (Hz), Pitch variability (Hz), Mean center (Hz) of gravity Formants, Speaking rate, Syllables per breath group, Jitter, Jitter ppq5, Shimmer, Shimmer apq3, Shimmer apq5, HNR, ComParE_2016	Acc: 75.12%, Sens: 85%
[76]	2021	Disease Identification: COPD	Private—49 subjects (11 COPD exacerbation, 9 Stable COPD, 29 Healthy)	Smartphone	LDA, SVM	Duration, the four formants, mean gravity center, some measures of pitch and intensity, openSMILE, eGeMAPS, # of words read out loud, duration of file	*p* < 0.01
[77]	2021	Disease Identification: COVID-19	Coswara—Dataset 1: 1040 subjects (965 non-COVID), Dataset 2: 990 subjects (930 non-COVID)	Smartphone	LR, MLP, RF	39-dimensional MFCCs + Δ and ΔΔ coeff., window size of 1024 samples, window hop size = 441 samples	Dataset 1 - RF: Average AUC: 70.69%, Dataset 2 - RF: Average AUC: 70.17%
[78]	2020	Disease Identification: COVID-19	Israeli COVID-19 collection—88 subjects (29 positive, 59 negative)	Smartphone	Transformer, SVM	Mel spectrum transformation	/z/: F1: 81%, Prec: 82%, counting: F1: 80%, Prec: 80%, /z/, /ah/: F1: 79%, Prec: 80%, /ah/: F1: 74%, Prec: 83%, cough: 58%, Prec: 72%
[79]	2021	Disease Identification: COVID-19, Asthma	COVID-19 sounds—1541 Respiratory Sounds	Mobile App, Web App–Smartphone, Various Microphones	light-weight CNN	MMFCC, EGFCC and Data De-noising Auto encoder	COVID-19/non-COVID-19 + breath + cough: Acc: 89%, Asthma/non-asthma + breath + voice Acc: 84%
[80]	2022	Disease Identification: Asthma	Private—8 subjects (100 normal, 321 Wheezing, 98 Striding, 73 Rattling sounds)	N/A	DQNN, Hybrid machine learning	IWO, Signal Selection: EHS algorithm	Spec: 99.8%, Sens: 99.2%, Acc: 100%
[81]	2022	Disease Identification: Asthma	18 patients—300 respiratory sounds, 10 types of breathing	N/A	DENN	IWO Algorithm for Asthma Detection & Forecasting	Spec: 99.8%, Sens: 99.2%, Acc: 99.91%
[82]	2020	Disease Identification: Asthma	Private—95 subjects (47 asthmatic, 48 healthy)	Various Microphones	SVM	ISCB using openSMILE, SET A: 5900 features, SET B: 6373 features, MFCC	/oU/ All feature groups: Acc: 74%
[13]	2020	Disease Identification: COVID-19	Private–240 acoustic data—60 normal, 20 COVID-19 subjects	Smartphone	LSTM (RNN)	Spec. Centroid, Spec. roll-off, ZCS, MFCC (+ΔΔ)	Cough: F1: 97.9% acc: 97%, breathing: F1: 98.8% acc: 98.2%, voices: F1: 92.5% acc: 88.2%
[83]	2020	Disease Identification: Asthma	88 recordings: 1957 segments (65 Severe resp. distress, 216 Asthma, 673 Mild resp. distress)	Smartphone	LIBSVM	Acoustic features: Interspeed 2010 Paralinguistic Challenge, 38 LLDs and 21 functionals	Acoustic Features: Acc: 86.3%, Sens: 85.9%, Spec: 86.9%
[84]	2021	Disease Identification: Asthma	Private—30 subjects	N/A	RDNN	Discrete Ripplet-II Transform	Proposed EAP-DL: Acc: 86.3%, Sens: 85.9%, Spec: 86.9%
[85]	2022	Symptom Identification: Voice Alteration	OPJHRC Fortis hospital in Raigarh—Cohort, not specified	Various Microphones	*K*-NN, SVM, LDA, LR, Linear SVM, etc.	Formant Frequencies, Pitch, Intensity, Jitter, Shimmer, Mean Autocorrelation, Harmonic to Noise ratio, Noice to Harmonic ration, MFCC, LPC	Decision Tree *K*-fold: Acc: 90% Sen: 90% Spec: 90%
[86]	2019	Symptom Identification: Voice Alteration	Private—Cohort n.s.	Various Microphones	Pretrained from Intel OpenVIVO and TensorFlow	Not specified, however models are vision based	N/A
[87]	2021	Disease Identification: COVID-19	Coswara, Cambridge DB-2—4352 Web App users, 2261 Android App users	Smartphone	SVM	MFCC	Acc: 85.7%, F2: 85.1%

Note. **ML models:** SVM = Support Vector Machine; *K*-NN = *K*-Nearest Neighbors; DT = Decision Trees; RF = Random Forest; NN = Neural Network; D-CNN = Deep Convolutional Neural Network; MLP = Multilayer Perceptron; NB = Naive Bayes; IWO = ImprovedWeed Optimization; DENN = Differential Evolutionary Neural Network; RBF model = Radial Basis Function model; LR = Linear Regression; LWL = Locally Weighted Regression (or Lowess); LDA = Linear Discriminant Analysis. **Data Processing Methods:** MFCCs = Mel-Frequency Cepstral Coefficients; CIF = Cochleagram Image Features; EGFCC = Enhanced-Gamma-tone Frequency Cepstral Coefficients; MMFCC = Modified Mel-frequency Cepstral Coefficients; IWO = Improved Weed Optimization; EHS = Effective Hand Strength; ISCB = Improved Standard Capon Beamforming; LPC = Linear Predictive Coding; FF = Fundamental Frequency; ZCS = Zero Crossing Rate. **Metrics:** Acc = Accuracy; Sens = Sensitivity; Spec = Specificity; Prec = precision; AUC = Area Under Curve.

**Table 4:** Open source Datasets.

Reference	Title	Description	Provider	Suitable for Respiratory Disease Classification	Suitable for Cough Detection
[92]	COUGHVID	Over 25,000 crowdsourced audio recordings: Cough—a wide range of participant ages, genders, geographic locations, and COVID-19 statuses	Embedded Systems Laboratory (ESL), EPFL, Lausanne, Switzerland	X	✓
[49]	COVID-19 Sounds	53,449 audio recordings, over 552 h in total: 3 Cough, 3–5 Breathing, 3 Speech of users reading a specific sentence	University of Cambridge	X	✓
[39]	Coswara	2747 audio recordings: Breathing, Coughing, Talking—Crowdsourced dataset (not clinically validated)	Indian Institute of Science (IISc), Bangalore	✓	✓
[69]	Respiratory Sound Database (RSDB)	920 audio recordings: Crackles or/and Wheezes - Digital stethoscopes and microphones, each recording is expertly annotated	Department of Informatics Engineering, University of Coimbra, Portugal and School of Medicine, Aristotle University of Thessaloniki, Greece	✓	X
[40]	Corp	168 h of 9969 audio recordings: Cough—42 different patients with respiratory diseases	MARI Lab, Tongji university	X	✓
[37]	Virufy	Combination of Coswara & COUGHVID audio recordings: Cough—COVID-19 positive/negative	The Covid Detection Foundation (California nonprofit corporation)	X	✓
[93]	COVID-19 and Pulmonary Abnormalities	1734 COVID-19 spectrogram images of respiratory sounds: 795 Crackles, 322 Wheezes, 1143 Normal.	Indian Institute of Science, PES University, M S Ramaiah Institute of Technology, Concordia University	✓	X
[94]	Tos-COVID	Audio recordings: Cough	Gov. of Buenos Aires city	X	✓
[95]	SPRSound: Open-Source SJTU Paediatric Respiratory Sound Database	2683 audio recordings and 9089 audio events: Respiratory Symptoms/Sounds—292 participants.	Shanghai Jiao Tong University and Shanghai Children’s Medical Center (SCMC)	✓	X
[96]	HF_Lung	Audio recordings: Lung Sounds/Symptoms—Used for developing automated inhalation, exhalation, and adventitious sound detection algorithms	Taiwan Smart Emergency and Critical Care (TSECC) and Taiwan Society of Emergency and Critical Care Medicine (TSECCM)	✓	X
[97]	ESC-50	2000 audio recordings: Environmental, Various, Cough—Labeled collection suitable for benchmarking methods of sound classification	Warsaw University of Technology, Warsaw, Poland	∼	✓
[98]	AudioSet	2,084,320 10-s audio recordings: Environmental, Various, Cough, Respiratory Symptoms—Expanding ontology, 632 human-labeled audio event classes, drawn from YouTube videos	Sound and Video Understanding teams, Google LLC	∼	✓
